# Effect of Inulin-Type Fructans on Body Composition, Carbohydrate Metabolism and Energy Expenditure in Patients with Psoriasis: Results of INGUTSKIN Randomized Controlled Trial

**DOI:** 10.3390/nu18121843

**Published:** 2026-06-08

**Authors:** Karolina Bieglecka, Paulina Katarzyna Kęszycka, Joanna Czerwińska, Krzysztof Pastuszak, Ewa Lange, Agnieszka Owczarczyk-Saczonek, Urszula Krupa-Kozak

**Affiliations:** 1 Chemistry and Biodynamics of Food Team, InLife Institute of Animal Reproduction and Food Research, Polish Academy of Sciences, 10-683 Olsztyn, Poland; k.bieglecka@pan.olsztyn.pl; 2 Department of Dietetics, Institute of Human Nutrition Sciences, Warsaw University of Life Sciences—SGGW, 02-787 Warsaw, Poland; paulina_keszycka@sggw.edu.pl (P.K.K.); ewa_lange@sggw.edu.pl (E.L.); 3 Department of Dermatology, Sexually Transmitted Diseases and Clinical Immunology, The University of Warmia and Mazury, 10-229 Olsztyn, Poland; joanna.czerwinska@uwm.edu.pl (J.C.); agnieszka.owczarczyk@uwm.edu.pl (A.O.-S.); 4 Department of Algorithms and System Modelling, Gdańsk University of Technology, 80-233 Gdańsk, Poland; krzpastu@pg.edu.pl; 5 Department of Translational Oncology, Medical University of Gdańsk, 80-309 Gdańsk, Poland; 6 Center of Biostatistics and Bioinformatics, Medical University of Gdańsk, 80-211 Gdańsk, Poland

**Keywords:** chronic skin inflammation, prebiotic, BMI, anthropometric indices, bioelectrical impedance analysis, resting energy expenditure

## Abstract

Introduction: Patients with psoriasis, a non-contagious, chronic, systemic inflammatory skin disease, often suffer from carbohydrate metabolism disorders, which can exacerbate systemic inflammation. Methods: This randomized, double-blind, placebo-controlled trial (RCT) evaluated the effects of chicory-derived inulin-type fructans (ITFs) on body composition, energy expenditure, and carbohydrate metabolism in patients with mild psoriasis (PS), using a healthy control (*n* = 32) group as a baseline reference. PS participants (*n* = 56) were randomized to receive 15 g/day of ITFs (*n* = 29) or a placebo (*n* = 27) for 8 weeks. Body composition using bioelectrical impedance analysis (BIA), carbohydrate metabolism (fasting glucose, insulin, glycated hemoglobin, Oral Glucose Tolerance Test (OGTT)) and resting energy expenditure (resting metabolic rate (RMR), oxygen consumption (VO_2_), carbon dioxide production (VCO_2_)) via indirect calorimetry were determined. Results: At baseline, patients with PS had significantly (*p* < 0.01) higher body mass index and visceral fat levels, and abnormal levels of selected parameters of carbohydrate metabolism, compared with healthy controls. Following the intervention, the ITFs group maintained stable fasting glucose levels, while the placebo group showed an undesirable increase (Δ_glucose_ = +5.7 mg/dL; *p* < 0.01). The energy expenditure analysis revealed a significant treatment effect on RMR and VO_2_ parameters (*p* < 0.01), with a decrease in the placebo group (*p* < 0.01) while remaining stable in the prebiotic group. There was no significant effect of the intervention on body composition and anthropometric parameters. Conclusions: Patients with PS exhibit a higher metabolic risk than healthy controls. Although no significant changes were observed within the intervention groups, the deterioration of metabolic indices in the placebo group may indicate an unfavorable effect of the maltodextrin. Consequently, the stabilization of parameters in the prebiotic group should be interpreted with great caution. This RCT was registered at ClinicalTrials.gov (NCT05971992).

## 1. Introduction

Psoriasis (PS) is a non-contagious, chronic, systemic inflammatory skin disease characterized by the presence of red, scaly, and often painful psoriatic plaques, resulting from, among others, excessive keratinocyte proliferation influenced by the IL-23/Th17 axis [1,2]. The consequence of activation of inflammatory cells and production of inflammatory cytokines is the onset of systemic inflammation, leading to faster development of atherosclerosis and, consequently, the emergence of metabolic disorders [3]. Approximately 2–3% of the global population is affected, with around 1.7% in Poland, and the number of detected cases is rising annually [4,5]. About 90% of patients are diagnosed with psoriasis vulgaris. Psoriasis is often comorbid with cardiovascular disease, obesity, metabolic syndrome, psoriatic arthritis, celiac disease, or depression, among other conditions [1]. The treatment is determined by PS type, severity, and comorbidities. In severe cases, systemic treatment (pharmacotherapy and biologic therapies) is used in combination with phototherapy; however, these methods are often associated with side effects [6]. Psoriasis is triggered by many external factors, such as infections, stress, medications, and diet [7]. An appropriate dietary strategy is part of a holistic approach to patient care. When there are no contraindications, a diet rich in omega-3 fatty acids, monounsaturated fats, complex carbohydrates, and dietary fiber may promote skin health and reduce the PASI (Psoriasis Area and Severity Index) [8].

Inulin is an indigestible carbohydrate classified as a dietary fiber, composed of D-fructofuranose units with different degrees of polymerization. It is naturally present in many plants, like onions or garlic, but a significant amount is found in chicory root. Inulin acts as a prebiotic by being fermented in the large intestine by gut microbiota. This process promotes the growth of beneficial bacteria and stimulates the production of short-chain fatty acids (SCFAs). As a result, inulin can improve nutrient absorption, relieve constipation, and offer various other health benefits [9,10,11].

Inulin-type fructans (ITFs) may contribute to weight loss and a reduction in body fat, including visceral fat, by creating a feeling of satiety and through SCFAs [12,13]; however, there are also reports indicating no effect on body composition [11,14]. While the exact mechanisms remain to be elucidated, SCFAs are known to influence energy storage by modulating the gut microbiota, directly affecting peripheral tissues, and regulating the metabolism of anorexigenic hormones. Furthermore, a systemic deficiency in SCFAs may actively contribute to the exacerbation of psoriasis. For instance, butyrate is critically responsible for the differentiation of regulatory T cells (Tregs) in both the intestines and the skin. A reduction in these cells impairs immune system regulation, thereby fueling the systemic inflammatory cascade that worsens psoriasis symptoms, including cutaneous inflammation [15,16,17].

Weight loss, particularly a reduction in body fat, enhances tissue insulin sensitivity, which in turn leads to changes in glucose metabolism, and insulin and glycated hemoglobin levels. Patients with PS often suffer from carbohydrate metabolism disorders, which can exacerbate systemic inflammation [18]. Therefore, improving these metabolic parameters through dietary supplementation with ITFs could provide valuable support in managing the metabolic issues faced by this patient group.

Changes in body weight and fat mass are accompanied by alterations in whole-body metabolism and energy expenditure. Resting metabolic rate (RMR) is strongly associated with body composition, and its stabilization may support the maintenance of weight loss outcomes [19,20]. For this reason, assessing RMR using indirect calorimetry by measuring oxygen consumption (VO_2_) and carbon dioxide production (VCO_2_) allows evaluation of the effect of ITFs supplementation on energy balance in patients with PS.

According to our knowledge, no previous studies have investigated the administration of inulin or ITFs, or any other prebiotics, in individuals with PS. The only reports on the effect of inulin on PS come from animal studies [21,22]. Taking into consideration the safety of use and the confirmed beneficial effects of prebiotics, including inulin, in patients with other skin diseases [23], this study aimed to evaluate the clinical effects of supplementation with chicory-derived ITFs vs. placebo on body composition, energy expenditure, and carbohydrate metabolism in adult patients with mild PS, compared to a healthy control group as a baseline reference for metabolic and anthropometric parameters.

## 2. Materials and Methods

### 2.1. Study Design

This study was designed as a single-center, randomized, double-blind, controlled, clinical trial (RCT) in which PS patients were allocated to either a prebiotic group or a placebo group receiving ITFs or maltodextrin for 8 weeks [24]. Furthermore, a control group of healthy subjects who did not receive any supplementation was established as a reference for metabolic and anthropometric parameters.

### 2.2. Ethics

The protocol of this RCT was approved by the Bioethics Committee of the Faculty of Medical Sciences at the University of Warmia and Mazury in Olsztyn (Resolution No. 1/2023, 19 January 2023). All procedures were conducted in accordance with the ethical principles set out in the Declaration of Helsinki [25]. The trial was registered at ClinicalTrials.gov (NCT05971992) [26].

### 2.3. Study Participants

**Figure 1:**
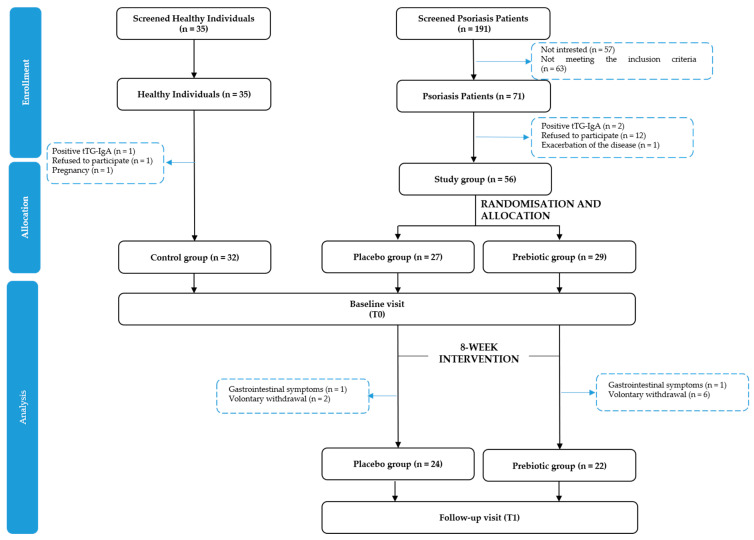
Study design and participants’ recruitment flow design according to CONSORT [27].

The detailed recruitment procedure (Figure 1) was described previously [24].

Briefly, study participants were recruited by an experienced dermatologist from among adult inpatients and outpatients with PS at the Clinic and Department of Dermatology, Sexually Transmitted Diseases, and Clinical Immunology of the Municipal Hospital Complex in Olsztyn (Poland). As part of the eligibility procedure, all participants underwent measurements of body weight and height. A clinical interview was also conducted regarding a history of gluten-related disorders (celiac disease, gluten sensitivity, or allergy to gluten and/or wheat protein), followed by testing for serum IgA antibodies against tissue transglutaminase (anti-tTG IgA) [6]. Participants had full access to all measurement results, and the detection of any abnormalities at this stage resulted in exclusion from the study. Ultimately, out of 191 screened candidates, 56 patients with mild PS (Psoriasis Area and Severity Index (PASI) < 10) [28,29] who met the specific inclusion/exclusion criteria (Table 1) were enrolled into the study group (Figure 1). Of these, 46 participants (82%) completed the study (Figure 1).

Moreover, 32 healthy subjects who met the eligibility criteria (Figure 1) were recruited to the control group.

All participants provided written informed consent to participate in the study, to the processing of their personal data, and were informed of their right to withdraw from the study at any time without consequences.

### 2.4. Randomisation

An external bioinformatician conducted the randomisation. A covariate-adaptive randomisation procedure for two treatments was applied to balance the treatment allocations across the selected covariates (gender, age and body mass index (BMI)) using R software (https://www.r-project.org/). Patients were randomly assigned to the prebiotic group (*n* = 29) or a placebo group (*n* = 27) (Figure 1).

### 2.5. Intervention

In this RCT, a prebiotic chicory-derived inulin-type fructans (ITFs) (Orafti^®^Synergy1, Beneo, Tienen, Belgium) and maltodextrin (Nutridex, OMNIA EUROPE SA, Medgidia, Romania) were used as supplements in the 8-week nutritional intervention. Supplements were packaged into identical moisture-proof, unlabeled sachets (EDPOL Food & Innovation Sp. z o.o., Łomża, Poland) containing 7.5 g of powder. Packages of sachets containing either prebiotic or placebo were prepared for the entire intervention period.

To ensure blinding, the packages were distributed to study participants by a person not directly involved in the study. The trial employed a double-blind design, in which the researchers, laboratory personnel, and participants were unaware of which participant received the package of sachets containing the prebiotic and which the placebo.

Participants were asked to consume the supplement 15–20 min before a meal by dissolving the powder in a lukewarm liquid. To minimize the risk of adverse events, the first week of intervention was the adaptation week, during which participants consumed only one sachet (7.5 g) of the supplement daily. Then, from week 2 until the end of the intervention, participants consumed two sachets daily (15 g total). To monitor treatment compliance, participants recorded the daily intake of the supplement, as well as any adverse reactions occurring during the trial in the observation questionnaire provided to each PS participant (in Polish). At the end of the intervention, participants returned the completed questionnaires, which were used to assess the compliance, with a minimum threshold of 80% required. The dosage of the supplement and the duration of the intervention were based on the previous studies [30,31].

### 2.6. Assessments

This RCT included two check-up visits for the study group: baseline visit (T0) before starting the intervention and follow-up visit (T1) after its completion. The control group underwent a one-time examination for comparative purposes (Figure 1). During visits T0 and T1, anthropometric indices and body composition were determined, and metabolic rate evaluation was performed in all participants after an overnight fast. In addition, an oral glucose tolerance test (OGTT) was performed using capillary blood samples collected from the fingertip at 0, 15, 30, 45, 60, 90, and 120 min after glucose consumption using glucometers and diagnostic strips (Contour Plus Ascensia, Ascensia Diabetes Care Poland Sp. z o. o., Warsaw, Poland).

#### 2.6.1. Anthropometric Indices and Body Composition

Body height was measured without shoes in a standing position using a SECA stadiometer (Seca, Hamburg, Germany) with an accuracy of 0.1 cm. To assess central obesity and metabolic risk, waist (at the midpoint between the lowest rib and the top of the hip bone at the end of normal expiration) and hip circumferences (at the widest part of the buttocks) were measured with a standard metric tape to calculate the waist-to-hip ratio (WHR).

Body weight and composition were evaluated via bioelectrical impedance analysis (BIA) using the Tanita BC-418 analyzer (Tanita Corporation, Tokyo, Japan). To ensure accuracy and safety, participants were required to empty their bladder before measurement, remove their shoes, wear light clothing, and remove any metal jewelry. The device was calibrated for adults, accounting for a 0.5 kg clothing weight allowance. Parameters determined included total body mass, BMI, fat mass (FM), visceral fat level (VFL), fat-free mass (FFM), predicted muscle mass (PMM), total body water (TBW), and phase angle (PhA).

#### 2.6.2. Metabolic Rate Analysis

Resting energy expenditure (RMR) was determined using an indirect static calorimetry with an open-circuit Cortex MetaMax 3B mobile device (CORTEX Biophysik GmbH, Leipzig, Germany). Measurements were performed on an empty stomach, in a supine position, under conditions of psychological and thermal comfort (constant room temperature of 22 ± 1 °C), after prior (no less than 5 min) adaptation to the conditions of the study, to achieve a coefficient of variation in measurements ≤ 10%. The ventilated mobile breath-by-breath system was used to determine Resting Metabolic Rate (RMR), RMR/BM (Body Mass (BM)), RMR/BSA (Body Surface Area (BSA)), oxygen consumption (VO_2_), carbon dioxide production (VCO_2_), percentage of energy derived from carbohydrate oxidation (CHO (%)), percentage of energy derived from fat oxidation (Fat (%)), percentage of energy derived from protein oxidation (Protein (%)), respiratory quotient (RQ).

#### 2.6.3. Carbohydrate Metabolism Analysis

Venous blood (6 mL) was collected using a serum vacuum tube (Greiner Bio-One GmbH, Kremsmünster, Austria) after an overnight fast to determine the fasting glucose and insulin using standard procedures at the outpatient clinic of the Municipal Hospital in Olsztyn (Poland). Insulin resistance was estimated using the Homeostasis model assessment index (HOMA-IR), using the following formula: (Insulin [µlU/mL] × Glucose [mg/dL]/405) [32].

A glycated hemoglobin (HbA1c) was determined using a commercially available enzyme-linked immunosorbent assay (ELISA) according to the manufacturer’s instructions (Human Glycated Hemoglobin A1c (HbA1c) ELISA Kit, Biorbyt, Cambridge, UK, detection range 15.63–1000 µg/mL).

#### 2.6.4. Oral Glucose Tolerance Test

The OGTT was performed under medical supervision. Participants were instructed to fast and avoid intense physical activity, alcohol, and analgesics the day before the test. Capillary blood glucose was measured at baseline (0 min) and at 15, 30, 45, 60, 90, and 120 min after the ingestion of a water solution of 75 g of glucose. Samples were obtained using disposable lancets (Accu-Chek Safe-T-Pro, Roche Diabetes Care, Poland) and analyzed with a glucometer (Contour Plus, Ascensia Diabetes Care Poland Sp. z o. o., Warsaw, Poland). The test results were conducted to determine the area under the curve with respect to ground (AUC_G_), using the trapezoidal rule for independent of the total number of measurements and repetitions proposed by Pruessner et al. [33]:

AUC_G_ = ∑i = 1*n* − 1(m_(i+1)_ + m_i_)∙t_i_2,

t_i_—individual time distance between measurements,
m_i_—individual measurement,
*n*—total amount of measures.

### 2.7. Statistical Analysis

The sample size was calculated for the original primary endpoint of the INGUTSKIN trial, defined as the between-group difference in PASI score change from baseline (T0) to the end of the 8-week intervention (T1). According to the study protocol [24], 25 evaluable participants per randomized arm were required to detect a moderate-to-large standardized between-group difference in PASI change of approximately 0.8 with 80% power and a two-sided alpha level of 0.05. Allowing for an anticipated 20% dropout rate, the planned recruitment target was 30 participants per randomized arm. The present analyses of body composition, carbohydrate metabolism, and energy expenditure were secondary and were not powered separately; therefore, these results were interpreted as exploratory.

Continuous variables are presented as mean ± standard deviation (SD), and categorical variables as number and percentage. Descriptive summaries were calculated from all available non-missing observations for each variable and time point. Baseline comparisons between patients with psoriasis and healthy controls were used to characterize the study groups only, because the healthy control group was not randomized or matched and was not included in longitudinal treatment-effect analyses. These baseline comparisons were performed using the Wilcoxon rank-sum test for continuous variables and Fisher’s exact test for categorical variables.

Longitudinal treatment effects among randomized PS participants were analyzed using linear mixed-effects models fitted separately for each intervention outcome. Each model included group, time, and the group × time interaction as fixed effects, with participant as a random intercept. The group × time interaction was the main treatment-effect contrast, testing whether the change from baseline differed between the prebiotic and placebo arms. Models were fitted using all available non-missing T0 and T1 observations for the given outcome, without imputation. Fixed effects were evaluated using Type III tests, and model assumptions were assessed using residual-versus-fitted and Q-Q plots.

Within-group placebo and prebiotic effects were assessed using paired Wilcoxon signed-rank tests in the predefined completer set used for delta analyses, defined as participants with available BMI/body-composition data at both T0 and T1. For outcomes with additional missing values, only participants within this completer set with paired T0 and T1 values for the corresponding outcome contributed to the test. Therefore, the number of observations used for T0 and T1 descriptive summaries may differ from the paired sample size used for within-group *p*-values. Delta values shown in figures and heatmaps were calculated as T1 minus T0 in the same predefined completer set. Spearman’s rank correlation was used to assess associations between delta values.

All tests were two-sided, with *p* < 0.05 considered statistically significant for nominal analyses. Because multiple secondary outcomes were analyzed and no formal multiplicity adjustment was applied, *p*-values were interpreted cautiously and only as exploratory evidence. Analyses were performed using R Statistical Software (version 4.3.2.) or GraphPad Prism version 10.0.3 (275) for Windows (GraphPad Software, LLC, Boston, MA, USA). 

## 3. Results and Discussion

The baseline characteristics of PS patients of the study group (*n* = 56) and healthy subjects of the control group (*n* = 32) are summarized in Table 2. The average age of PS patients was 42 years, and their mean BMI was 25.6 (Table 2). The gender distribution was uneven, with a higher representation of males (55%) compared to females (45%). At baseline, the average score for PASI was 3.5, indicating a mild form of the disease [28,29]. The PS patients exhibited higher BMI (*p* < 0.001), VFL (*p* < 0.001), and FM compared to the control group. In contrast, their FFM and PMM were lower than those of the control group (Table 2).

The results of the present study align with previous research and clearly showed differences in body composition between patients with mild PS and healthy individuals, indicating increased BMI and VFL as risk factors for metabolic syndrome (Mets) development [34,35,36]. A population-based, cross-sectional study in the United Kingdom confirmed that PS patients face a 22% increased risk of developing metabolic syndrome (MetS) compared to healthy controls [37]. Moreover, the authors indicated that PS severity affected the degree of association, with the MetS seen in 32% of patients with mild PS, 36% with moderate PS, and 40% with severe PS. This resulted from a direct impact of the PS characteristics, which is recognized as a chronic systemic inflammatory condition rather than just a skin disease, with inflammation driving adipose tissue dysfunction [38].

### 3.1. The Effect of the Nutritional Intervention on Anthropometric Indices and Body Composition of the Study Participants

The effect of the nutritional intervention with prebiotic ITFs vs. placebo (maltodextrin) on anthropometric indices and body composition of PS patients is presented in Table 3. The 8-week intervention revealed no statistically significant between-group treatment effects, as evidenced by the Treatment Effect. However, exploratory within-group analysis within the prebiotic cohort (Prebiotic Effect) indicated subtle, non-significant shifts that may suggest a potential stabilizing direction in FM (*p* = 0.060), PMM (*p* = 0.076) and FFM (*p* = 0.070). In contrast, the placebo group remained entirely stable across these biological markers.

Our study did not detect significant changes in body composition in PS patients as a consequence of nutritional intervention. While these within-group trends within the ITF cohort are noteworthy, they must be interpreted strictly as observational, given the lack of a true statistical separation from the placebo group.

Dewulf et al. [12], in a double-blind, placebo-controlled intervention study on obese women, showed that ITFs had no impact on BMI and waist/hip ratio but tended to decrease fat mass. Li et al. [39], in a randomized, double-blind, placebo-controlled clinical trial, demonstrated that 4-week supplementation with 10 g/day of inulin reduced blood glucose (mmol/L) at 1 and 2 h during OGTT in overweight/obese individuals but not in healthy subjects, while no changes were observed in the placebo group. Many studies and systematic reviews on prebiotic supplementation indicate their more pronounced impact in obese individuals, contrary to normal-weight or overweight populations, where a significant effect on body composition was not detected [40,41].

Not only the type of supplement but also the duration of the intervention may influence the obtained results. The 8-week supplementation period was a relatively short time for achieving a significant difference in body composition parameters. Le Bourgot et al. [42], in a RCT involving overweight individuals with prediabetes, demonstrated that 12 weeks of supplementation with short-chain fructooligosaccharides (scFOS; 8 g/day) compared to placebo led to improvements in BMI, FFM, and FM compared to the placebo group (maltodextrin). Similar results were observed by Buhas et al. [43], in a 12-week, open-label, non-randomized clinical trial in patients with PS. They showed that compared to the control group, supplementation with a synbiotic (prebiotics: FOS, XOS, GOS, and probiotics Bacillus spp.; a total of 2 × 10^9^ CFU/day) led to improvements in disease severity (PASI), and BMI and FFM; however, the use of a complex intervention makes it impossible to distinguish the effects of prebiotics and probiotics. These observations suggest that a longer intervention might be necessary to reach the threshold of statistical significance.

### 3.2. Carbohydrate Metabolism

The results obtained showed differences in carbohydrate metabolism between PS patients and healthy subjects (Table 2). We detected substantial inter-individual variations in the baseline glucose level in PS patients. Compared to the control group, significantly higher fasting glucose, HbA1c and AUC values were detected in the PS group (*p* = 0.002; *p* = 0.008; *p* = 0.004, respectively). The carbohydrate profile, in particular blood glucose level, was near the upper limit of normal in both groups (according to Polish guidelines 70–99 mg/dL (3.9–5.5 mmol/L [44]) and HbA1c levels twice as high, suggesting an increased risk of insulin resistance and diabetes—components of metabolic syndrome—in individuals with PS [45,46].

**Figure 2:**
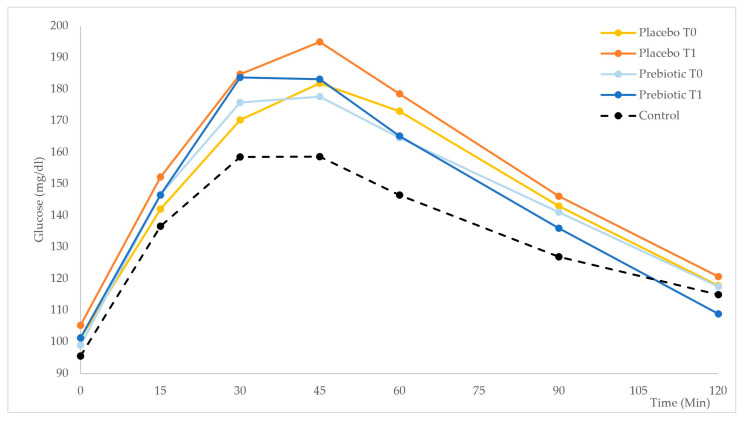
The visualization of the oral glucose tolerance test (OGTT-75) obtained from capillary fingertip blood in placebo and prebiotic groups before (T0) and after the intervention (T1), and in the control group.

The impact of dietary supplementation with prebiotic ITFs vs. placebo on carbohydrate metabolism is presented in Table 4 and Figure 2. Glucose levels in the placebo group increased significantly at T1 (+5.7 mg/dL; *p* = 0.006), while the prebiotic group showed no significant changes (+2.2 mg/dL; *p* = 0.177). However, the overall glucose tolerance, assessed via AUC values, did not reach any significance. The remaining parameters examined (insulin, HOMA-IR, HbA1c) showed no significant within-group changes or overall treatment effect.

Regarding the glycemic profile, the 8-week supplementation with ITFs appeared to support within-group glycemic stability, preventing the significant rise in fasting glucose that occurred in the placebo group. However, because the overall between-group interaction for fasting glucose did not reach statistical significance (*p* = 0.146), and these values increased significantly in the placebo group (*p* = 0.006), the data should be interpreted with caution. This difference can be attributed to the contrasting metabolic pathways of the two substances. While inulin is an indigestible fructose polymer with a negligible glycemic index, maltodextrin, used as the placebo, is a starch-derived polysaccharide that undergoes rapid enzymatic hydrolysis and is largely absorbed as glucose in the small intestine [47,48]. Crucially, maltodextrin exhibits a remarkably high glycemic index that is comparable to or even exceeds that of pure glucose, directly accounting for the rapid blood sugar spikes and the significant increase in fasting glucose observed in the placebo group [49]. Such acute elevations in blood sugar can induce excessive insulin release and precipitate potential metabolic disruptions, including oxidative stress, inflammation, and cellular damage, which are closely linked to long-term health problems such as insulin resistance, an increased risk of type 2 diabetes, and cardiovascular disease [50]. Consequently, the inherent metabolic activity of maltodextrin represents a major interpretative challenge in studies evaluating inulin-type fructans (ITFs), as its unfavorable impact on carbohydrate metabolism complicates the clean comparison with the active intervention group, rendering it a common yet imperfect choice for a placebo. The AUC was calculated to assess changes in the glycemic response following the intervention. Detected in the placebo group, an increase in AUC may indicate a deterioration of glucose tolerance over time, whereas this parameter remained stable in the prebiotic group, however without statistical significance. A study by Li et al. [39] showed that inulin reduces AUC in obese individuals, whereas in normal-weight individuals, these changes are often insignificant.

Our findings are consistent with observations in healthy individuals without baseline carbohydrate metabolism disorders, where ITF supplementation does not cause significant changes [15]. The opposite effect is observed in patients with already established prediabetes or type 2 diabetes, where ITF supplementation leads to an overall improvement in glycemic parameters [51,52]. Considering that the baseline results of carbohydrate metabolism obtained from PS participants were within the normal limits or did not differ from those of the healthy controls, it can be concluded that, in this case, the ITFs intervention has no effect on metabolic markers. It is worth noting that the observed AUC value (*p* = 0.052) reflects the unfavorable glucose increase within the placebo group rather than a metabolic change caused by the prebiotics. Because ITFs maintained glycemic stability while the active placebo caused a spike, the statistical distance between the groups was artificially reduced, meaning that a definitive differentiation remains to be validated in future trials.

### 3.3. Metabolic Rate

The PS group did not show any differences in metabolic rate parameters compared to healthy controls (Table 2).

**Figure 3:**
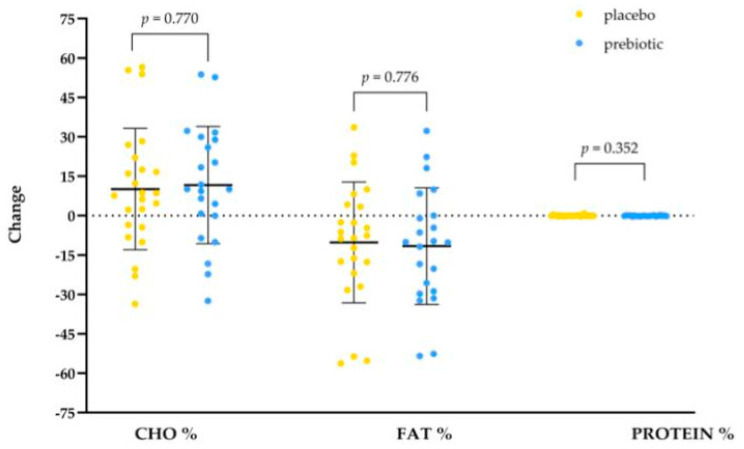
Mean changes (∆T1–T0) in selected metabolic rate parameters between the placebo and prebiotic groups after the nutritional intervention. CHO (%): percentage of energy derived from carbohydrate oxidation; Fat (%): percentage of energy derived from fat oxidation; Protein (%): percentage of energy derived from protein oxidation. Values are shown as mean ± SD. *p*-value: Wilcoxon signed-rank test.

The impact of nutritional intervention on the results of indirect calorimetry parameters in the studied groups is presented in Table 5. Analysis revealed significant changes in the treatment effect for RMR, RMR/BM ratio, RMR/BSA ratio, and VO_2_ (*p* = 0.009; *p* = 0.003; *p* = 0.005; *p* = 0.011, respectively). In the placebo group, these parameters significantly decreased at T1 (*p* = 0.005; *p* = 0.004; *p* = 0.005; *p* = 0.003, respectively), with no effect observed in the prebiotic group. Regarding substrate oxidation, both the placebo and prebiotic groups showed significant within-group changes in CHO (*p* = 0.049 vs. *p* = 0.046, respectively) and fat oxidation (*p* = 0.049 vs. *p* = 0.042, respectively). Despite these intra-group shifts, no significant treatment effect was observed, as further confirmed by the Delta analysis illustrated in Figure 3. No changes were also observed in RQ, VCO_2_ and protein oxidation.

Our results reveal a difference in the metabolic profiles of the two groups. Specifically, the placebo group experienced a significant decrease in RMR, which represents an unfavorable shift, as a reduction in basal energy expenditure typically indicates a slowing of metabolic activity. The intervention with ITFs maintained baseline stability in these parameters. To verify that these differences were not merely due to variations in the participants’ body composition, the RMR/BM and RMR/BSA ratios were also analyzed. The fact that these indices (accounting for body mass and surface area) showed analogous trends demonstrates that the maintenance of metabolic rate was not only a secondary effect of weight stabilization. It is worth noting, however, that while the increase in metabolic rate following supplementation is more pronounced in obese individuals, this effect was moderate in our group, resulting in a milder effect due to BMI restrictions.

The marked decrease observed in the placebo group may have several underlying mechanisms. This decrease could potentially reflect the natural, time-dependent course of the underlying condition in the PS study participants, whereby progressive systemic factors or disease progression may spontaneously suppress resting energy expenditure over time. This change could also be due to seasonal changes, spontaneous lifestyle changes during the study, or unexpected metabolic responses to the placebo vehicle itself. Given that ITF did not actively increase these parameters but rather maintained baseline values, these interpretations remain speculative and should be treated with caution.

The gas exchange parameters are another indicator of metabolic rate. A significant decrease in VO_2_ in the placebo group indicates a reduction in energy expenditure and a slowing of metabolic processes in that group. Another aspect of the analysis involved the assessment of substrate oxidation, based on the Respiratory Quotient (RQ), calculated as the ratio of carbon dioxide produced to oxygen consumed [53]. The RQ in both groups at baseline indicated balanced nutrient oxidation, whereas at T1 a shift toward greater carbohydrate oxidation was observed. Interestingly, although prebiotic ITFs had a significant effect on the proportions of macronutrients utilized compared with the placebo group, this did not significantly affect overall metabolic rate. These observations lead to the assumption that it remains difficult to definitively determine whether ITFs exerted a genuine stabilizing effect on resting energy expenditure or if the variance between the cohorts was primarily driven by an unfavorable metabolic shift induced by the placebo.

### 3.4. PASI Score

The PASI score is the most commonly used measure of the severity of PS, allowing for an assessment of the extent and intensity of skin lesions. Scores on this scale range from 0 to 72 points, with a higher score indicating a more severe course of the disease. The assessment is based on an analysis of four anatomical areas of the body: the head (10%), upper extremities (20%), trunk (30%), and lower extremities (40%). In each of these areas, three clinical parameters are assessed: erythema, induration, and desquamation on a scale from 0 to 4, and the score is then adjusted by the estimated percentage of affected skin area [28,29].

**Figure 4:**
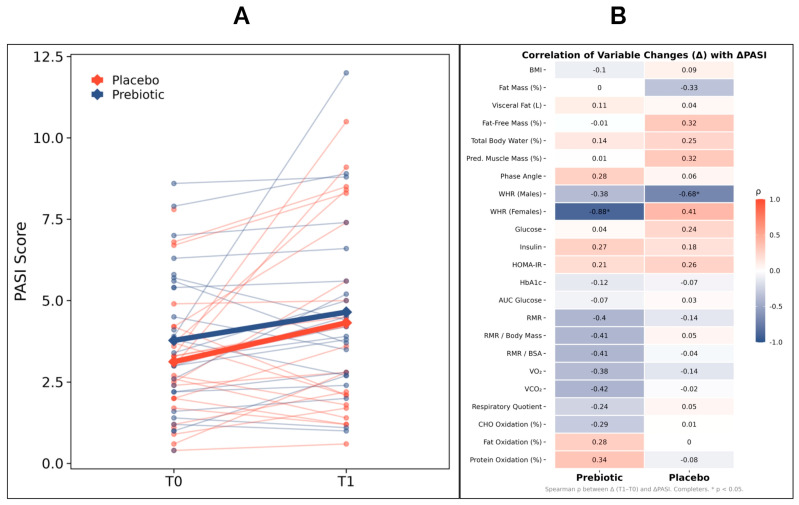
Impact of the dietary intervention on psoriasis severity and its metabolic correlates in a study group. (**A**) Individual and mean PASI score changes from baseline (T0) to the end of treatment (T1). (**B**) Spearman correlation heatmap between changes (∆) in physiological variables and changes (∆) in PASI score.

A comparative analysis of PASI changes was conducted for all PS participants. The characteristics of symptom severity at time points T0 and T1, along with statistical analysis of intergroup differences, are summarized in Figure 4. Both groups showed a worsening of PASI scores, which reached statistical significance only in the placebo group (3.1 ± 1.8 to 4.3 ± 3.0; *p* = 0.021) compared to the prebiotic group (3.8 ± 2.1 to 4.6 ± 2.7; *p* = 0.082); however, despite this increase, the condition remained classified as mild psoriasis with individual fluctuations and mean scores illustrated in Figure 4A. A Spearman correlation heatmap between changes (∆) in physiological variables and changes (∆) in PASI score (Figure 4B) showed a strong inverse correlation between WHR Females and PASI in the prebiotic group, while an inverse correlation between WHR Males and PASI was detected in the placebo group.

### 3.5. Strengths and Limitations

The main strength of this study is its pioneering nature, as to our knowledge, this is the first RCT to evaluate the effects of ITF supplementation on the metabolic rate parameters and body composition specifically in patients with PS. By employing an RCT design, this study meets the highest standards of clinical research due to its rigorous evaluation of the intervention. The use of indirect calorimetry allowed for a precise determination of how prebiotics influence metabolic homeostasis beyond simple anthropometric measurements. Furthermore, the inclusion of a healthy control group at baseline provided a crucial reference point, highlighting the significant metabolic disparities and increased health risks inherent to the psoriatic population. The reliability of the observed protective trends is further confirmed by the strictly controlled study protocol and the inclusion of a placebo group as a reference point.

Despite these strengths, some limitations should be acknowledged. First, the 8-week intervention period may have been too short to detect statistically significant changes in body composition and certain metabolic parameters. As suggested by the literature, these changes often require a longer duration to become apparent.

Second, although the sample size was determined by a power analysis to ensure statistical validity, the overall number of participants may not have been sufficient to allow for complex subgroup analyses. Moreover, while patients were instructed to maintain their usual diet, the absence of a strictly controlled, standardized meal plan throughout the study could have introduced confounding variables. However, it is possible that the differences observed between the placebo and prebiotic groups were due to unmeasured dietary differences between the groups rather than the intervention itself. For instance, an increased or uneven intake of dietary fiber, omega-3 fatty acids, or polyphenols could have independently modulated host metabolism and gut microbiota activity, thereby confounding the observed outcomes and masking the true effects of the intervention [54,55].

Third, certain methodological approaches limit the direct comparison of our findings with standard clinical benchmarks and other clinical trials. HbA1c levels in this study were determined using a commercially available quantitative enzyme-linked immunosorbent assay (ELISA) kit. This specific immunoassay measures the absolute concentration of glycated hemoglobin in the sample, yielding results expressed in µg/mL rather than the standard clinical percentages (%) or mmol/mol used in routine whole-blood laboratory diagnostics. Consequently, while these measurements are perfectly reliable for evaluating internal within-group changes and between-group differences over time, they cannot be directly extrapolated to conventional clinical reference ranges. The interpretation of these results should focus strictly on the relative shifts over time (T0 vs. T1) and the statistical comparison between the studied groups.

Finally, we realize that choosing maltodextrin as a placebo is not ideal, as it may induce various modifications in the gut microbiome configuration and immunological factors [56]. However, maltodextrin is a common choice in inulin studies, and in our RCT, we relied on guidance from the literature and examples from other studies [30,57] and our previous experiences [58].

Future research should consider extending the intervention period to at least 12 weeks to more accurately assess the impact of ITF on anthropometric parameters and body composition. It would also be beneficial to conduct gender-stratified analyses in a larger cohort, as hormonal and metabolic differences between men and women may significantly influence the response to prebiotic supplementation. Furthermore, future studies could focus more specifically on overweight and obese patients, excluding individuals with normal body weight, to better capture the potential of ITF in groups where metabolic disturbances are more pronounced.

## 4. Conclusions

A comparison of the PS individuals with healthy controls revealed differences in body composition and carbohydrate metabolism. Due to their less favorable metabolic profile, PS patients were shown to be at greater risk of developing insulin resistance/type 2 diabetes or metabolic syndrome.

The 8-week intervention with ITFs did not significantly affect body composition, carbohydrate metabolism, or resting energy expenditure parameters in PS patients. Instead, the statistically relevant variances observed between the study arms were driven by a metabolic deterioration within the placebo group, which exhibited increased fasting glucose levels alongside a significant decrease in RMR and VO_2_.

Our results indicate that ITF supplementation remained metabolically neutral in the study cohort, while the use of maltodextrin calls into question its status as a widely accepted placebo standard in inulin studies. The observed discrepancies between groups should, in fact, be attributed to adverse metabolic changes induced by the placebo vehicle itself, rather than to the active, stabilizing, or therapeutic effects of the prebiotic. Further long-term studies are needed to clearly determine the independent metabolic impact of ITF in this population, with consideration given to the use of a different type of placebo and/or the inclusion of a non-supplemented group.

## Figures and Tables

**Table 1:** Inclusion and exclusion criteria.

Category	Control Group	Study Group
Inclusion criteria		
Age 18–65 years	✓	✓
BMI * < 30 kg/m^2^	✓	✓
Unrestricted diet	✓	✓
tTG-IgA seronegative	✓	✓
Written informed consent	✓	✓
Mild psoriasis (PASI < 10)	-	✓
Exclusion criteria		
Chronic or acute inflammatory disease (other than psoriasis) **	✗	✗
Current anti-psoriatic treatment (systemic or biologic)	-	✗
Antibiotics within 14 days before sampling	✗	✗
Probiotic, prebiotic, and/or synbiotic supplements intake within 4 weeks before sampling	✗	✗
Pregnancy, breastfeeding	✗	✗
Surgery within 6 months before sampling	✗	✗

* BMI (Body Mass Index); PASI: Psoriasis Area and Severity Index ** particular diabetes, gastrointestinal disease, cardiovascular complications, heart, kidney and liver failure, thyroid disease, cancer.

**Table 2:** Baseline characteristics of the study cohorts.

	Control Group (*n* = 32)	Study Group (*n* = 56)	*p*-Value
Sex (F/M)	19/13	25/31	0.268
Age (y)	32 ± 8	42 ± 8	<0.001
BMI (kg/m^2^)	23.4 ± 2.7	25.6 ± 3.0	<0.001
FM (%)	23.9 ± 5.3	26.6 ± 6.2	0.096
VFL	4.0 ± 3.0	7.0 ± 3.1	<0.001
FFM (%)	76.1 ± 5.3	73.4 ± 6.2	0.095
TBW (%)	50.6 ± 4.4	50.6 ± 4.7	0.838
PMM (%)	72.3 ± 5	69.7 ± 5.9	0.097
PhA (°)	5.7 ± 0.5	6.0 ± 0.7	0.082
WHR (Males)	0.91 ± 0.05	0.89 ± 0.06	0.346
WHR (Females)	0.77 ± 0.05	0.79 ± 0.07	0.323
Glucose (mg/dL)	81.1 ± 8.7	88.5 ± 10.1	0.002
Insulin (µIU/mL)	10.9 ± 3.6	11.4 ± 8.4 (*n* = 55) *	0.143
HOMA-IR	2.2 ± 0.8	2.5 ± 1.9 (*n* = 55) *	0.613
HbA1c (µg/mL)	123 ± 48	258 ± 226 (*n* = 55) *	0.008
AUC_G_ (mg/dL·h)	271 ± 27 (*n* = 31) **	300 ± 44	0.004
RMR (kcal/d)	1778 ± 300	1812 ± 395	0.606
RMR/BM (kcal/d/kg)	24.9 ± 3.3	23.7 ± 4.3	0.125
RMR/BSA (kcal/d/m^2^)	952 ± 110	948 ± 163	0.811
VO_2_ (L/min)	0.26 ± 0.04	0.26 ± 0.06	0.657
VCO_2_ (L/min)	0.22 ± 0.04	0.23 ± 0.06	0.411
RQ	0.85 ± 0.06	0.87 ± 0.09	0.188
CHO (%)	47.6 ± 15.6	53.5 ± 21	0.167
Fat (%)	47.7 ± 15.6	41.9 ± 20.9	0.169
Protein (%)	4.6 ± 0.1	4.6 ± 0.1	0.103

WHR: Waist-Hip Ratio; BMI: Body Mass Index; FM: Fat Mass; VFL: Visceral Fat Level; PMM: Predicted Muscle Mass; FFM: Fat Free Mass; TBW: Total Body Water; PhA: Phase Angle; HOMA-IR: Homeostasis Model Assessment—Insulin Resistance; HbA1c: Glycated Hemoglobin; AUC_G_: area under the curve with respect to ground; RMR: Resting Metabolic Rate; BM: Body Mass; BSA: Body Surface Area; VO_2_: oxygen consumption; VCO_2_: carbon dioxide production; CHO (%): percentage of energy derived from carbohydrate oxidation; Fat (%): percentage of energy derived from fat oxidation; Protein (%): percentage of energy derived from protein oxidation; RQ: respiratory quotient. Values are shown as the mean ± standard deviation (SD). All psoriasis vs controls *p*-value: Wilcoxon signed-rank test; Sex: Fisher’s exact. * Insufficient blood sample collected for testing. ** Refuse for OGTT (Oral glucose tolerance test).

**Table 3:** Body composition and anthropometric changes in the studied groups.

	Placebo		Placebo Effect	Prebiotic		Prebiotic Effect	Treatment Effect
	T0	T1	*p*-Value	T0	T1 *	*p*-Value	*p*-Value
BMI (kg/m^2^)	25.8 ± 3.1	26.2 ± 3.1	0.107	25.4 ± 2.9	25.6 ± 3.0	0.374	0.962
FM (%)	26.8 ± 6.5	26.7 ± 7.0	0.254	26.5 ± 6.1	25.8 ± 5.9	0.060	0.604
VFL	7.0 ± 3.4	7.0 ± 3.6	0.777	7.0 ± 2.9	7.0 ± 2.8	0.374	0.254
PMM (%)	69.5 ± 6.2	69.7 ± 6.7	0.277	69.9 ± 5.8	70.5 ± 5.6	0.076	0.656
FFM (%)	73.2 ± 6.5	73.3 ± 7.0	0.290	73.5 ± 6.1	74.2 ± 5.9	0.070	0.654
TBW (%)	50.4 ± 4.5	50.0 ± 4.9	0.060	50.9 ± 5.0	50.9 ± 4.8	0.668	0.835
PhA (°)	6.01 ± 0.68	5.95 ± 0.72	0.584	5.97 ± 0.69	5.95 ± 0.69	0.473	0.996
WHR Male	0.90 ± 0.06	0.91 ± 0.05	0.970	0.88 ± 0.05	0.88 ± 0.06	0.685	0.749
WHR Female	0.79 ± 0.07	0.80 ± 0.07	0.970	0.79 ± 0.07	0.81 ± 0.07	0.844	0.782

WHR: Waist-Hip Ratio; BMI: Body Mass Index; FM: Fat Mass; VFL: Visceral Fat Level; PMM: Predicted Muscle Mass; FFM: Fat Free Mass; TBW: Total Body Water; PhA: Phase Angle; Values are shown as the mean ± standard deviation (SD). Placebo/Prebiotic Effect: paired Wilcoxon (within-group, completers). Treatment Effect: Group × Time interaction from LMM (ITT). * Missing data of body composition measurement in prebiotic group at T1 (*n* = 21).

**Table 4:** Carbohydrate metabolism changes in the studied groups.

	Placebo		Placebo Effect	Prebiotic		Prebiotic Effect	Treatment Effect
	T0	T1	*p*-Value	T0	T1	*p*-Value	*p*-Value
Glucose (mg/dL)	90.8 ± 11.9	96.5 ± 10.8	0.006	86.3 ± 7.5	88.5 ± 7.1	0.177	0.146
Insulin (µlU/mL)	11.7 ± 7.1	11.6 ± 4.2	0.684	11.0 ± 9.5 (*n* = 28) *	10.5 ± 6.3	0.393	0.983
HOMA-IR	2.6 ± 1.6	2.8 ± 1.2	0.290	2.4 ± 2.2 (*n* = 28) *	2.3 ± 1.4	0.243	0.718
HbA1c (µg/mL)	269 ± 241	282 ± 174	0.747	248 ± 215 (*n* = 28) *	314 ± 209	0.179	0.417
AUC_G_ (mg/dL∙h)	302 ± 38	316 ± 43	0.070	299 ± 50	298 ± 47	0.140	0.052

HOMA-IR: Homeostasis Model Assessment—Insulin Resistance; HbA1c: Glycated Hemoglobin; AUC_G_: area under the curve with respect to ground; Values are shown as the mean ± standard deviation (SD). Placebo/Prebiotic Effect: paired Wilcoxon (within-group, completers). Treatment Effect: Group × Time interaction from LMM (ITT). * Insufficient blood sample collected for testing.

**Table 5:** Metabolic rate changes in the studied groups.

	Placebo		Placebo Effect	Prebiotic		Prebiotic Effect	Treatment Effect
	T0	T1	*p*-Value	T0	T1	*p*-Value	*p*-Value
RMR (kcal/d)	1881 ± 372	1710 ± 354	0.005	1748 ± 412	1822.0 ± 290	0.865	0.009
RMR/BM (kcal/d/kg)	24.7 ± 4.2	22.1 ± 3.7	0.004	22.8 ± 4.3	23.7 ± 3.3	0.945	0.003
RMR/BSA (kcal/d/m^2^)	990 ± 149	894 ± 134	0.005	908.6 ± 168.4	946.3 ± 100.9	0.917	0.005
VO_2_ (L/min)	0.27 ± 0.05	0.24 ± 0.05	0.003	0.25 ± 0.06	0.26 ± 0.04	0.983	0.011
VCO_2_ (L/min)	0.24 ± 0.05	0.22 ± 0.05	0.110	0.22 ± 0.07	0.23 ± 0.04	0.647	0.137
RQ	0.87 ± 0.09	0.89 ± 0.06	0.053	0.87 ± 0.10	0.89 ± 0.07	0.159	0.892
CHO (%)	54.7 ± 22.0	64.2 ± 18.1	0.049	52.5 ± 20.3	61.3 ± 20.2	0.046	0.988
Fat (%)	40.7 ± 21.9	31.2 ± 18.1	0.049	42.9 ± 20.3	34.1 ± 20.1	0.042	0.995
Protein (%)	4.6 ± 0.1	4.6 ± 0.2	0.726	4.6 ± 0.1	4.6 ± 0.1	0.517	0.389

RMR: Resting Metabolic Rate; BM: Body Mass; BSA: Body Surface Area; VO_2_: oxygen consumption; VCO_2_: carbon dioxide production; CHO (%): percentage of energy derived from carbohydrate oxidation; Fat (%): percentage of energy derived from fat oxidation; Protein (%): percentage of energy derived from protein oxidation; RQ: respiratory quotient. Values are shown as the mean ± standard deviation (SD). Placebo/Prebiotic Effect: paired Wilcoxon (within-group, completers). Treatment Effect: Group × Time interaction from LMM (ITT).

## Data Availability

Data presented in the present study are available in the RepOD repository [59].

## Abbreviations

The following abbreviations are used in this manuscript:

Anti-tTG IgA	Iga antibody against tissue transglutaminase
AUC_G_	Area under the curve with respect to ground
BIA	Bioelectrical impedance analysis
BM	Body mass
BMI	Body mass index
BSA	Body surface area
CHO (%)	Percentage of energy derived from carbohydrate oxidation
Fat (%)	Percentage of energy derived from fat oxidation
FFM	Fat-free mass
FM	Fat mass
HbA1c	Glycated hemoglobin
HOMA-IR	Homeostasis model assessment index
ITF	Inulin-type fructans
OGTT	Oral glucose tolerance test
PASI	Psoriasis Area and Severity Index
PhA	Phase angle
PMM	Muscle mass
Protein (%)	Percentage of energy derived from protein oxidation
PS	Psoriasis
RCT	Randomized, controlled trial
RMR	Resting metabolic rate
RQ	Respiratory quotient
SCFA	Short-chain fatty acids
TBW	Total body water
VCO_2_	Carbon dioxide production
VFL	Visceral fat level
VO_2_	Oxygen consumption
WHR	Waist-to-hip ratio
